# Subdiffraction localization of a nanostructured photosensitizer in bacterial cells

**DOI:** 10.1038/srep15564

**Published:** 2015-10-23

**Authors:** Pietro Delcanale, Francesca Pennacchietti, Giulio Maestrini, Beatriz Rodríguez-Amigo, Paolo Bianchini, Alberto Diaspro, Alessandro Iagatti, Barbara Patrizi, Paolo Foggi, Monserrat Agut, Santi Nonell, Stefania Abbruzzetti, Cristiano Viappiani

**Affiliations:** 1 Dipartimento di Fisica e Scienze della Terra, Università di Parma, Viale delle Scienze 7A, 43124 Parma, Italy; 2 Dipartimento di Bioscienze, Università di Parma, Viale delle Scienze 11A, 43124 Parma, Italy; 3 NEST, Istituto Nanoscienze, Consiglio Nazionale delle Ricerche, Piazza San Silvestro 12, 56127 Pisa, Italy; 4 Fondazione Istituto Italiano di Tecnologia, Via Morego, 30, 16163 Genova, Italy; 5 LENS (European Laboratory for Nonlinear Spectroscopy) Via N. Carrara 1, Sesto Fiorentino, Florence 50019, Italy; 6 INO (Istituto Nazionale di Ottica), Largo Fermi 6, Florence 50125, Italy; 7 Dipartimento di Chimica, Università di Perugia, via Elce di Sotto 8, Perugia, 06123 Italy; 8 Institut Quimic de Sarrià, Universitat Ramon Llull, Via Augusta 390, 08017 Barcelona, Spain

## Abstract

Antibacterial treatments based on photosensitized production of reactive oxygen species is a promising approach to address local microbial infections. Given the small size of bacterial cells, identification of the sites of binding of the photosensitizing molecules is a difficult issue to address with conventional microscopy. We show that the excited state properties of the naturally occurring photosensitizer hypericin can be exploited to perform STED microscopy on bacteria incubated with the complex between hypericin and apomyoglobin, a self-assembled nanostructure that confers very good bioavailability to the photosensitizer. Hypericin fluorescence is mostly localized at the bacterial wall, and accumulates at the polar regions of the cell and at sites of cell wall growth. While these features are shared by Gram-negative and Gram-positive bacteria, only the latter are effectively photoinactivated by light exposure.

Antibacterial treatment based on photosensitization relies on the combined action of an otherwise nontoxic molecule (called photosensitizer, PS), visible light, and oxygen to produce cytotoxic effects by the photoinduced generation of reactive oxygen species (ROS)^1^,^2^,^3^. This method has proved to be valuable for the treatment of localized microbial infections, effectively acting on several classes of microbial pathogens, without inducing insurgence of photoresistant species even after multiple treatments^4^,^5^. The molecular basis for the photoinduced cellular damage is in many cases the formation of singlet oxygen (^1^O_2_)^6^, a non-radical, electronically-excited form of the dioxygen molecule that is highly reactive against a vast array of cellular components ranging from membrane lipids to proteins and nucleic acids^1^,^2^,^7^. It is generally accepted that the photosensitization of ^1^O_2_ must occur in the close vicinity of the target cells in order to be able to induce photooxidative damage to cellular components, since the short lifetime of this ROS prevents interactions at distances exceeding a few hundred nanometers from the site of photosensitization^7^. Thus, the localization of the PS largely dictates where photodamage will first be inflicted.

This type of information is generally retrieved from indirect experimental evidence, based on the efficiency of cell inactivation, photophysical measurements, and laborious analysis of cell damage products^2^,^3^,^8^,^9^.

It is therefore highly desirable to identify the cellular distribution of the PS directly from spectroscopic markers with high spatial resolution in order to understand which cellular components are most likely to be damaged by the photosensitized ROS. Several PS molecules exhibit more or less intense fluorescence emission that may be exploited to identify the location of the PS inside the cell. However, given the very small size of targeted bacterial cells, on the order of magnitude of the resolution of confocal microscopes, a conventional fluorescence imaging approach is not suitable for assessing localization of the dye inside bacteria. Fluorescence microscopy with sub-diffraction resolution appears to be the method of choice to address this issue^10^,^11^,^12^ yet it has not been applied so far to the study of bacteria photoinactivation.

We have thus explored the possibility to exploit the intense fluorescence emission of a naturally occurring PS molecule, hypericin (Hyp), to identify its distribution inside living bacteria using a STimulated Emission Depletion (STED) microscopy approach. To the best of our knowledge this approach has not yet been reported for any PS molecule.

In a STED microscope, the excitation beam is spatially overlaid with a laser beam inducing stimulated emission (STED beam), which has a doughnut-shaped focal pattern featuring a “zero”-intensity point in its center. Thus, the STED beam inhibits fluorescence emission except at the center of the focus. At saturating intensity of the STED beam, the fluorescence emission is confined to a volume with subdiffraction size along the lateral direction^13^.

Hyp is a natural product, structurally belonging to the chemical class of naphthodianthrones, which is found in plants from the genus Hypericum, e.g., St. John’s Wort^14^,^15^. St. John’s Wort infusions are used as herbal aids for depression and Hyp has also been successfully utilized as an antiviral^16^,^17^, antibacterial^18^,^19^,^20^, and antifungal agent^21^.

While Hyp is readily dissolved in ethanol or dimethylsulfoxide (DMSO), where it shows sharp absorption bands, intense and structured fluorescence emission (quantum yield *Φ*_F_ = 0.35 in ethanol)^9^,^15^, and sensitizes singlet oxygen with high quantum yield (*Φ*_Δ_ = 0.32 in ethanol^22^, 0.39 ± 0.01 in methanol^23^, and 0.33 in DMSO^24^), it is essentially insoluble in water, since it forms aggregates that are very weakly fluorescent^25^ and do not lead to appreciable singlet oxygen photosensitization. Thus, nanocarriers are needed for the solubilization of Hyp in aqueous solutions, in which the photosensitizing properties of Hyp are preserved. We have recently shown that the protein apomyoglobin (apoMb) can be usefully exploited as a biocompatible nanocarrier for Hyp since it preserves its fluorescence, photodynamic properties and bacterial photoinactivation ability^3^,^26^,^27^. The inherent fluorescence emission of Hyp-apoMb is a fundamental prerequisite for the application of the photoactive drug in superresolution fluorescence imaging.

## Results

### Evidence of stimulated emission by Hyp-apoMb from femtosecond pump-and-probe transient absorbance

**Figure 1:**
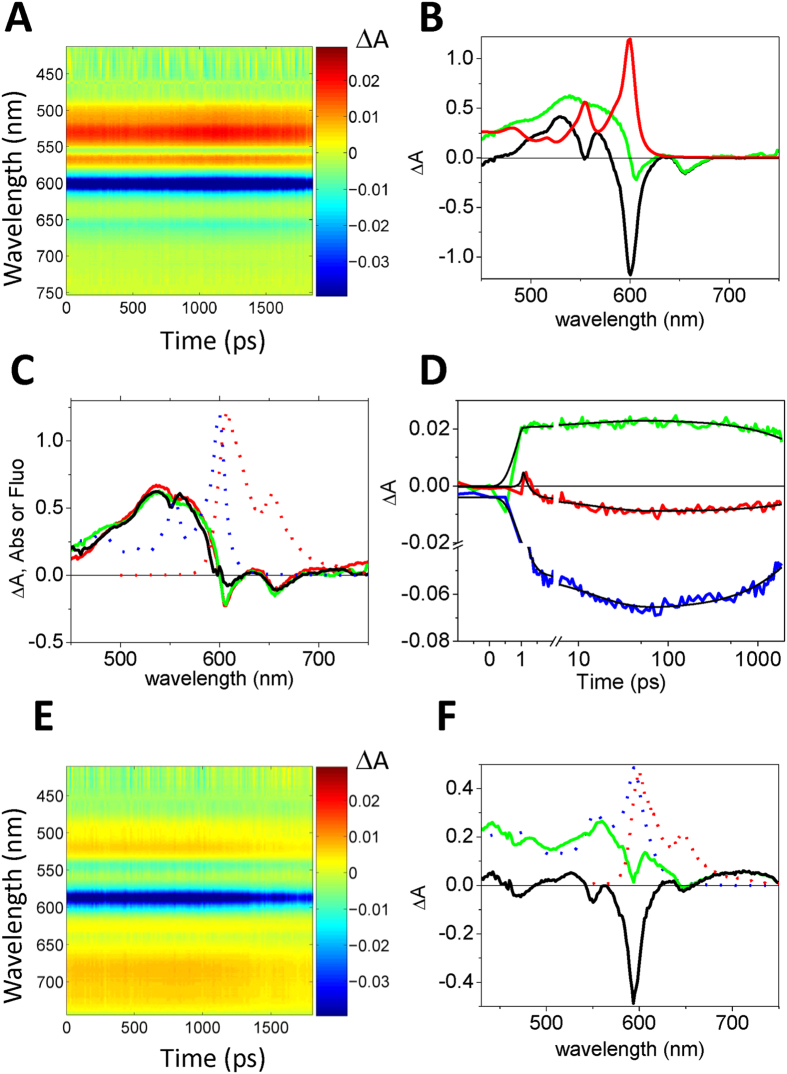
Detection of stimulated emission in femtosecond pump-and-probe experiments. (**A**) Transient absorption time/wavelength plot after femtosecond excitation at 400 nm for Hyp (120 μM) in DMSO. (**B**) The steady state absorption spectrum (red solid line) is used to remove the ground state bleaching from the raw transient absorption spectra (black line). This allows to retrieve corrected transient absorption spectra (green line). The example corresponds to the transient spectrum at 100 ps delay. (**C**) Corrected transient absorption spectra for Hyp (120 μM) in DMSO at 3 ps (black), 10 ps (red), and 100 ps (green). The dotted lines correspond to steady state absorption (blue) and fluorescence emission (red). (**D**) Absorption changes after 400 nm excitation of Hyp (120 μM) in DMSO at selected wavelengths, reflecting excited state absorption (521 nm, green), ground state bleaching (592 nm, blue), and stimulated emission (647 nm, red). Black solid lines are the result of a global analysis performed on the single traces using a sum of three exponential decay functions. (**E**) Transient absorption time/wavelength plot after femtosecond excitation at 400 nm for Hyp-apoMb (120 μM Hyp, 530 μM apoMb) in PBS. (**F**) The steady state absorption spectrum (blue dotted line) is used to remove from the raw transient absorption spectra (black line) the ground state bleaching. This allows to retrieve corrected transient absorption spectra (green line). The sample curves corresponds to a 1000 ps delay. The dotted red line corresponds to steady state fluorescence emission.

Not all fluorophores are amenable to STED superresolution microscopy, especially if excited state absorption bands are present in the spectral region where excited depletion is performed^28^,^29^. Femtosecond pump-and-probe transient absorbance^30^ was therefore employed to assess the possibility of inducing stimulated emission from the excited state of Hyp^28^,^31^. Figure 1A reports the raw transient absorption time/wavelength plot after femtosecond excitation at 400 nm for Hyp in DMSO, where prominent features of ground state bleaching are evident at 600 nm, whereas excited state absorption gives rise to positive bands at 531 nm and 567 nm. Subtracting the ground state absorption from the transient absorption spectrum allows removing the contribution of ground state bleaching, as exemplified in Fig. 1B for the data collected at 100 ps delay.

Figure 1C shows transient spectra thus corrected for the ground state bleaching at selected delays (3 ps, 10 ps, and 100 ps) after excitation. The corrected transient absorption spectra show broad excited state absorption features, peaked at roughly 550 nm and extending to 600 nm. No significant excited state absorbance is observed above 600 nm. Actually, increased light transmission (appearing as negative absorbance changes with peaks at 606 nm and 656 nm) is detected instead, which nicely correlates with steady state fluorescence emission (607 nm e 654 nm) and is thus attributed to stimulated emission by the probe pulse.

Time course of the measured kinetics are reported in Fig. 1D at selected wavelengths, corresponding to excited state absorption (521 nm, green), ground state bleaching (592 nm, blue), and stimulated emission (647 nm, red). We have chosen to monitor the stimulated emission at the red edge of the spectrum to avoid self absorption at ≈600 nm by the highly concentrated Hyp solution. Global analysis affords a biphasic kinetics characterized by lifetimes of ≈10 ps and ≈6 ns. The first transient is attributed to excited-state intramolecular proton transfer^32^,^33^,^34^, whereas the second process corresponds to the singlet excited state decay, and is consistent with the measured fluorescence lifetime (Supporting Information Table S1)^25^.

Similar data were obtained on the Hyp-apoMb complex, although with a lower signal-to-noise ratio (Fig. 1E,F) owing to the lower concentration. Stimulated emission features can nevertheless be recognized at 594 nm and 650 nm, while excited state absorption bands appear broader and weaker in this case, extending to the near infrared. Excited state dynamics preserves the same features evidenced in DMSO (biphasic kinetics characterized by lifetimes of ≈7 ps and ≈3 ns), suggesting that, unlike other cases^35^,^36^, the protein matrix does not interfere with the intramolecular (lifetime ≈ 7 ps) proton transfer reaction.

### STED microscopy of bacteria loaded with Hyp-apoMb

Given the promising properties of the Hyp-apoMb excited states, we have tested the applicability of the nanocomplex to fluorescence imaging in Gram-positive (*Bacillus subtilis* and *Staphylococcus aureus*) and Gram-negative (*Escherichia coli*) bacteria using the STED approach. We have selected 715 nm as the wavelength of the STED beam, which is in resonance with a vibronic transition in the emission spectrum^26^.

**Figure 2:**
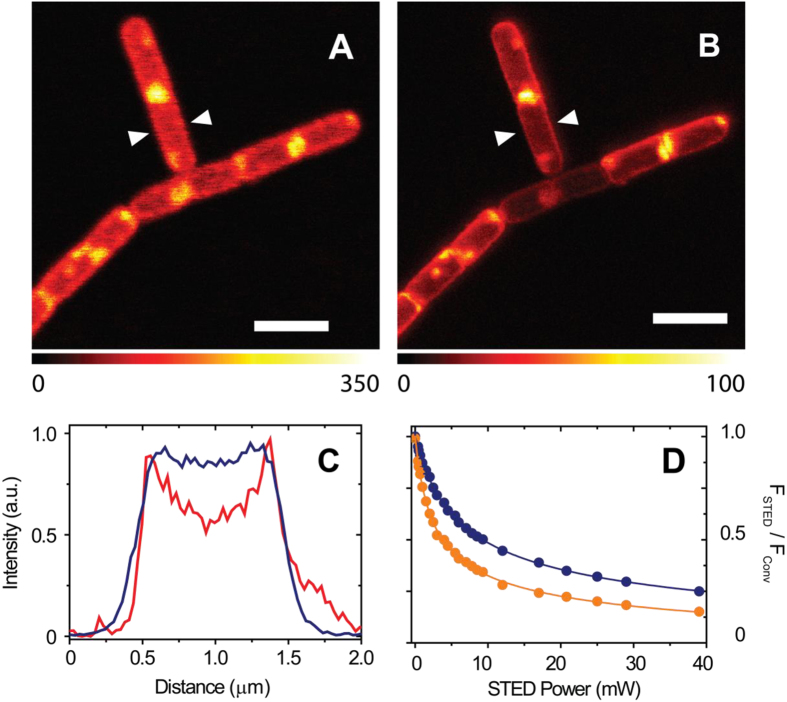
Improvement in resolution by STED microscopy. (**A**,**B**) Comparison between *B. subtilis* images collected with confocal microscopy (**A**) and with STED nanoscopy (STED power 30 mW, pixel dwell time 0.1 ms) (**B**). (**C**) The blue and the red intensity profiles were measured along the segment connecting the arrows in (**A**,**B**) respectively. Scale bars are 2.5 μm. (**D**) Fluorescence depletion curves for Hyp in DMSO (10 μM, orange circles; Is = 3.1 ± 0.1 mW, α = 0.10 ± 0.01 (see Supporting Information for definition of parameters) and Hyp-apoMb in PBS (10 μM Hyp, 30 μM apoMb, blue circles; I_s_ = 6.5 ± 0.1 mW, α = 0.14 ± 0.01) collected under excitation at 566 nm and detection at 605/70 nm. The STED beam was at 715 nm. Solid lines are the best fit to depletion functions (Equation 2).

Figure 2A shows that *B. subtilis* bacteria become highly fluorescent after being incubated with Hyp-apoMb, which proves that the protein carrier has delivered the fluorescent PS to the cells, preventing aggregation. When the STED laser is turned on, images undergo a remarkable improvement in resolution (Fig. 2B). This can be best appreciated by inspection of the fluorescence emission profile along a cross section of a cell, as reported in Fig. 2C. Sharp structural features are revealed, whose width is on the order of 90 nm, corresponding to the width of the bacterial wall, a complex structure formed by an outer rigid peptidoglycan layer and an inner cell membrane. The improvement in resolution is comparable to earlier reports for different dyes on the same type of bacteria^11^. With the current STED resolution it is still not possible to assess whether Hyp is localized on the external peptidoglycan layer or on the inner plasma membrane of these Gram-positive bacteria.

Figure 2D reports the depletion curves for the fluorescence emission at 605/70 nm under 566 nm excitation and depletion at 715 nm collected on Hyp in DSMO and Hyp-apoMb in PBS. While fluorescence emission can be depleted for both samples, saturation power is lower for Hyp in DMSO than for Hyp-apoMb in water. In both cases it was necessary to introduce an additive constant to properly describe the depletion curves, which may be taken as indication of a fraction of non-saturable fluorescence emission. The presence of excited state absorption of Hyp-apoMb in the spectral region of the STED beam (Fig. 1E,F) is possibly at the origin of the observed saturation parameters (higher saturation power and larger non-saturable fraction) for Hyp-apoMb.

**Figure 3:**
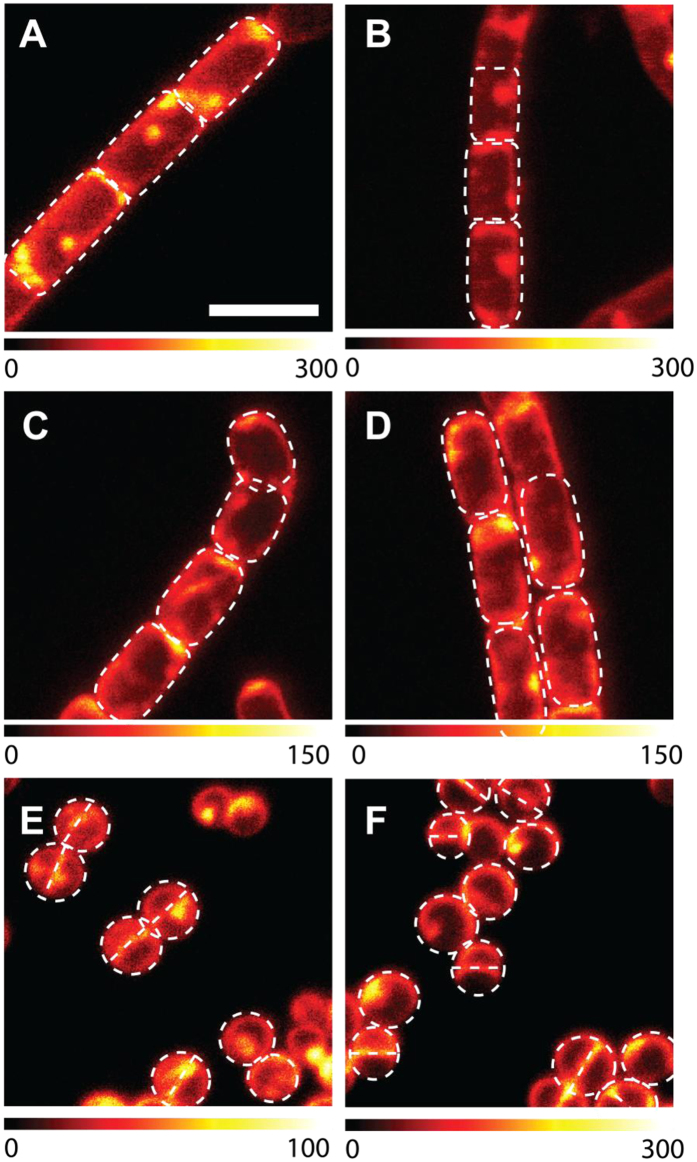
Localization of Hyp-apoMb in different types of bacteria. Selected STED images of *B. subtilis* (**A**,**B**), *E. coli* (**C**,**D**) and *S. aureus* cells (**E**,**F**) incubated with Hyp-apoMb (10 μM Hyp, 30 μM apoMb) collected under excitation at 566 nm and detection at 605/70 nm. The STED beam was at 715 nm, power 30 mW and dwell time 0.1 ms (**A**–**D**) and 0.05 ms (**E**,**F**). White dashed lines are intended as visual aid to guide the eye along the bacterial shape. Scale bar, 2 μm.

A closer look at Fig. 2B reveals that besides accumulating on the cell wall, Hyp appears to have high local concentrations also at specific cellular locations as judged from the intense fluorescence observed at the poles of the bacteria and at the central body of the cell. The fairly similar topology of accumulation points inside cells (Figs 2 and 3) suggests specific interactions with yet to be identified bacterial components, possibly located inside areas of MreB-dependent wall growth^37^, or cytoskeleton components^38^.

Images collected on *E. coli* suspensions incubated with Hyp-apoMb illustrate a distribution of the dye which is rather similar to the one observed for *B. subtilis*.

It is clear that while STED images conclusively show that the PS molecules accumulate mainly at the cell wall, the spatial resolution achieved in the present work does not yet allow the identification of the specific wall layer the photosensitizing construct is confined into, nor the identification of the molecular species it is bound to. Admittedly, the current spatial resolution obtained with Hyp-apoMb is comparable with the size of the cell wall, and it is therefore difficult to foresee a fine identification of the wall layer where the PS accumulates. Therefore, co-localization studies with specific bacterial wall components labeled with suitable fluorescent probes are expected to be of help in identifying more precisely the type of interactions occurring at the cell wall and the nature of the observed accumulation points.

The fluorescence emission by Hyp affords images (Figs 2 and 3) of dividing bacteria whose shape is consistent with the established division mechanisms. *B. subtilis* divides mainly by septation without constriction, resulting in its typically squared off poles (Figs 2 and 3A,B)^37^. As *B. subtilis* is Gram-positive and thus lacks an outer membrane, there is no need for coordinated constriction. On the contrary, dividing *E. coli* images (Fig. 3C,D) show round poles because the Gram-negative bacterium divides with a combination of septum formation and constriction of inner and outer membranes^39^. Growth and division of rod shaped bacteria such as *E. coli* is explained by sequential switching between two modes of growth. For newborn cells right after division, peptidoglycan is synthesized along the sidewall, leading to elongation of the cell to twice its original length. At the time of cell division, the synthesis apparatus switches from sidewall peptidoglycan synthesis to division septum synthesis^40^.

Unlike *B. subtilis* and *E. coli*, *Staphylococcus aureus* is spherical in shape. Cell wall growth occurs through a FtsZ dependent mechanism and septation separates two hemispheres (Fig. 3E,F)^37^. Areas of more intense fluorescence are observed at the septal region which appears thicker than the cell wall, in agreement with evidence derived from electron microscopy^41^. Interestingly, images collected on bacteria loaded with a concentrated Hyp solution in DMSO provide fluorescence distributions which are indistinguishable from those collected with Hyp-apoMb.

In spite of the very similar cellular distribution reported above, the consequences of light exposure on cell viability are remarkably different for bacterial types. While a light dose of 20 J/cm^2^ is enough to decrease the bacterial content by 5 log units for Gram-positive *S. aureus*, no sizeable effects are evident on Gram-negative *E. coli* (Supporting Information) The case of Gram-positive *B. subtilis* appears intermediate, with only a limited effect of light exposure. The different response of Gram-positive and Gram-negative bacteria to photodynamic therapy was reported in the literature for other PS molecules and has been correlated to the structure of the bacterial wall, consistent with our STED images that place Hyp mainly on the cell wall^18^. The cell wall of Gram-positive bacteria is composed by lipoteichoic and teichoic acids organized in multiple layers of peptidoglycan (30–100 nm)^42^, which permits PS penetration through the porous structure. At variance, Gram-negative bacteria have an intricate outer membrane in the cell wall that is impermeable to antimicrobial agents. It is composed by glycolipids in the outer leaflet, lipoproteins and β-barrel proteins, and a phospholipid bilayer in the inner leaflet to anchor those constituents, and peptidoglycan, for a thickness of 2–7 nm^41^,^42^. Further studies correlating PS cellular distribution with specific wall components may provide a rationale for distinguishing between the different response of Gram-positive and Gram-negative bacteria to PDT.

## Materials and Methods

Horse heart myoglobin was purchased from Sigma-Aldrich. Hypericin was obtained from HWI Analytik GmbH. All were used as received.

### ApoMb preparation

ApoMb was prepared from the holoprotein using standard biochemical procedures. The heme was removed by cold (−30 °C) acid acetone extraction from horse heart myoglobin^43^. The sample was washed with cold acetone and centrifuged several times, dried with pure nitrogen, and suspended in PBS buffer at pH 7.4. The suspension was then centrifuged, and the supernatant was spectroscopically checked to assess sample purity. The concentration of the ApoMb stock was calculated from the absorption at 280 nm (ε = 15,800 cm^−1^ M^−1^) and heme contamination was estimated from the absorption at 408 nm (ε = 179,000 cm^−1^ M^−1^)^44^. In all the preparations, heme contamination was typically 0.5% of the total protein content.

### Femtosecond Transient Absorption Spectroscopy (TAS)

The experimental set-up was described previously^30^,^45^. Briefly, it is based on an amplified Ti:sapphire laser system delivering pulses with time duration of ~100 fs. The output was frequency doubled for exciting the sample at 400 nm (pump energy = 0.15 μJ/pulse), while the time evolution of the excited protein was monitored by a second spectrally broad UV-visible pulse, the white continuum probe pulse, generated by focusing the fundamental beam on a calcium fluoride plate. The probe pulse is delayed with respect to the pump by means of a suitable optical line that allows to scan a time interval up to 2 ns after excitation. The repetition rate of the laser system was set at 100 Hz and the sample was kept under continuous stirring by means of a small magnet inside the cuvette (path length = 2 mm). All the measurements were carried out by setting the relative pump-probe polarization at the magic angle (54, 7°). The detection system consists of two linear CCD arrays (Hamamatsu S8377-256Q), coupled to a spectrograph (Jobin Yvon CP 140–1824) and controlled by a home-made front-end circuit. The signals were fed into a simultaneous analog-to-digital conversion board (Adlink DAQ2010) and data were acquired by means of a LabVIEW written computer program. By repeating the sequence as a function of the pump-probe delay, we were able to obtain the dynamical evolution of the transient absorbance ΔA(λ, t). Kinetics extracted at different wavelengths were fitted with a multiexponential response function, convoluted with a Gaussian instrumental function (FWHM = 160 fs). Furthermore, global analysis^46^ of kinetics recorded in the whole probed spectral range were applied. A sequential model was used to extract the spectral features of interest, associated with each transient.

### STED nanoscopy

Stimulated emission depletion (STED) nanoscopy has been performed using a custom made setup equipped with a supercontinuum pulsed laser source (ALP-710–745-SC, Fianium LTD, Southampton, UK). We selected the excitation wavelength by means of an AOTF, while the STED wavelength is predefined by the laser outputs, in particular the 715 nm output is in resonance with a vibronic transition in the emission spectrum^26^. The laser has a repetition frequency of 20 MHz and a pulse width of about 100 ps. In all the experiments we used 566 nm for excitation and 715 nm for STED. The doughnut shape of the STED beam is realized by a vortex phase plate (RPC photonics inc., Rochester, NY, USA). The beams are scanned on the sample by galvanometer mirrors (Till-photonics, FEI Munich GmbH, Germany), focused by a HCX PL APO CS 100 × 1.4 NA oil (Leica Microsystems, Mannheim, Germany) objective and fluorescence is collected by an avalanche photodiode (SPCM-AQRH-13-FC, Excelitas Technologies, Vaudreuil-Dorion, Quebec, Canada) in the spectral window 670–640 nm^47^.

In order to study the STED efficiency we measured the fluorescence depletion curve. For this experiment the STED beam is Gaussian shaped and we acquired several images of Hyp in solution varying the STED power. The resulting fluorescence depletion curve is defined as:


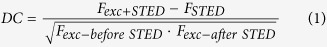


where *F*_*exc+STED*_ is the fluorescence measured in the presence of excitation and depletion beams, *F*_*STED*_ is the fluorescence measured in the presence of the depletion beam, *F*_*exc−before STED*_ and *F*_*exc−after STED*_ are the fluorescence measured in the presence of excitation beam before and after the measurement with STED beam, respectively.

The fluorescence depletion curves were described by the following equation^48^,^49^ and the data fitted accordingly:


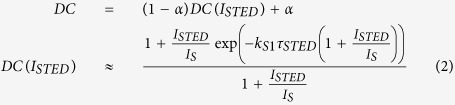


Where α is an additive constant describing the level of non-saturable fluorescence, I_STED_ is the STED beam power, I_S_ is the intensity of the STED beam at which the rate of stimulated emission equals the spontaneous decay, k_S1_ is the spontaneous decay rate and τ_STED_ is the STED pulse width.

## Additional Information

**How to cite this article**: Delcanale, P. *et al.* Subdiffraction localization of a nanostructured photosensitizer in bacterial cells. *Sci. Rep.* **5**, 15564; doi: 10.1038/srep15564 (2015).

## Supplementary Material

Supplementary Information
