# Supplementary material for: Subdiffraction localization of a nanostructured photosensitizer in bacterial cells
Source: Sci Rep. 2015 Oct 23;5:15564. doi: 10.1038/srep15564 (PMC4616064; doi:10.1038/srep15564)
Supplement: Supplementary Information [file srep15564-s1.pdf]

Supporting information to

Subdiffraction localization of a nanostructured photosensitizer in bacterial cells

Pietro Delcanale[†], Francesca Pennacchietti[‖], Giulio Maestrini[†], Beatriz Rodríguez-Amigo[♯], Paolo Bianchini[‖], Alberto Diaspro[‖], Alessandro Iagatti[&,%], Barbara Patrizi[&,%], Paolo Foggi[&,%,○], Monserrat Agut[♯], Santi Nonell[♯], Stefania Abbruzzetti[§,‡], Cristiano Viappiani[*,†,‡]

[†]Dipartimento di Fisica e Scienze della Terra, Università di Parma, Viale delle Scienze 7A, 43124 Parma, Italy

[§]Dipartimento di Bioscienze, Università di Parma, Viale delle Scienze 11A, 43124 Parma, Italy

[‡]NEST, Istituto Nanoscienze, Consiglio Nazionale delle Ricerche, Piazza San Silvestro 12, 56127 Pisa, Italy

[‖]Fondazione Istituto Italiano di Tecnologia, Via Morego, 30, 16163 Genova, Italy

[&]LENS (European Laboratory for Nonlinear Spectroscopy) Via N. Carrara 1, Sesto Fiorentino, Florence 50019, Italy

[%]INO (Istituto Nazionale di Ottica), Largo Fermi 6, Florence 50125, Italy

°Dipartimento di Chimica, Università di Perugia, via Elce di Sotto 8, Perugia, 06123 Italy

[♯]Institut Quimic de Sarrià, Universitat Ramon Llull, Via Augusta 390, 08017 Barcelona, Spain

Methods

Nanosecond laser flash photolysis

The nanosecond laser flash photolysis setup was described previously. [1, 2] Briefly, the second harmonic (532 nm) of a nanosecond Nd:YAG laser (Spectron laser) was used to excited Hyp solutions. Detection at right angle was achieved by means of the monochromatic output of a 150W Xe lamp coupled with a 25 cm monochromator (AMKO Gmnh). The detection wavelength was set to 520 nm and the transmitted light intensity was collected by a 5-stages photomultiplier (Applied Photophysics) through a 12-cm monochromator (LOT-Oriel). The residual stray light from the pump beam was rejected by a HR dichroic mirror optimized for high reflection at 532 nm (Omega Optical).

Fluorescence Correlation Spectroscopy

Fluorescence Correlation Spectroscopy (FCS) experiments were performed using a Microtime 200 from Picoquant, based on an inverted confocal microscope (Olympus IX70) and equipped with two SPADs (Single Photon Avalanche Diodes, Perkin Elmer) used in the cross correlation mode. Excitation was achieved by a 475 nm picosecond diode laser and fluorescence emission was collected through a bandpass filter centered at 650 nm and split with a 50/50 splitter between the two detection channels. The same setup allowed the determination of fluorescence decay lifetimes under the same experimental conditions used in the FCS experiments.

In order to achieve single molecule conditions while still preserving full binding of Hyp to apoMb, concentrations were [Hyp] = 10 nM and [apoMb] = 30 μM.

Singlet Oxygen Measurements

The quantum yield of singlet oxygen ($^1O_2$) production by Hyp dissolved in DMSO was determined by direct detection of $^1O_2$ phosphorescence at 1275 nm using a modified PicoQuant Fluotime 200 system. A diode-pumped pulsed Nd:YAG laser (FTSS355-Q, Crystal Laser, Berlin, Germany) working at 1 kHz repetition rate (λ_{exc}=532 nm, 1.2 μJ per pulse) was used for excitation; the NIR luminescence exiting from the sample was detected at 90° by a H9170-45 NIR-PMT module (Hamamatsu) working in photon counting mode and a NanoHarp 250 multichannel scaler (PicoQuant, Germany). Rose bengal (RB), tetrakis-(4-sulfonatophenyl)-porphine (TPPS) and 5,10,15,20-tetrakis(1-methyl-4-pyridinium)porphine tetra(*p*-toluenesulfonate) (TMPyP) were used as references. Time-resolved $^1O_2$ phosphorescence was detected under the same experimental conditions for Hyp and the references. All samples were dissolved in pure DMSO, air-equilibrated, and kept at a temperature of 25°C during the measurement.

The quantum yield of singlet oxygen production (Φ_Δ) was measured using the procedure described in reference[3] for homogeneous systems. For each photosensitizer, 5-6 samples were prepared at different concentrations, with absorbance in the range 0.01 – 0.1 at the λ_{exc}. The time-resolved phosphorescence signal, showing the typical rise and decay, was recorded for each sample and fitted with the equation:

$$S = S_0 \frac{\tau_\Delta}{\tau_\Delta - \tau_T}\left(e^{-t/\tau_\Delta} - e^{-t/\tau_T}\right) + y_0$$

where τ_T and τ_Δ are the lifetime of the photosensitizer triplet state and of $^1O_2$ respectively, y_0 is an offset due to instrument dark counts and S_0 is an instrumental quantity proportional Φ_Δ. For each photosensitizer, the S_0 values obtained were plotted against the sample absorption factor

$(1 - 10^{-A})$, where A is the sample absorbance at λ_{exc}. Data could well fitted using a linear model with zero intercept and Φ_Δ was then calculated comparing the slopes of Hyp and reference:

$$\Phi_{\Delta,Hyp} = \Phi_{\Delta,ref} \frac{s_{A,Hyp}}{s_{A,ref}}$$

where $s_{A,Hyp}$ and $s_{A,ref}$ are the slopes obtained respectively for Hyp and the reference photosensitizer. Since there is no well-established reference photosensitiser in DMSO, we hypothesized that the Φ_Δ values in water would hold in DMSO. This seems to be the case for the two porphyrins, which yielded a similar Φ_Δ value for Hyp in DMSO (Φ_Δ=0.28 ± 0.05 taking $\Phi_{\Delta,ref}$=0.74 for TMPyP and TPPS in aqueous solvents[4]). The Φ_Δ found is in good agreement with a previous value determined by laser-induced optoacoustic spectroscopy[5]. The value found using RB as reference was disregarded as unrealistically high (Φ_Δ=0.43 ± 0.05 taking $\Phi_{\Delta,ref}$=0.75 for RB in aqueous solvents[4]) since from the ratio of slopes it would be inferred that the Φ_Δ values of the porphyrins would be 1.15, which goes above the upper limit of 1.

Microbial growth conditions and photoinactivation of bacteria

We determine the photosensitizing efficiency of our photosensitizer on three bacterial strains: *E. coli* CECT101 and *S. aureus* CECT239 both obtained from the Spanish Type Culture Collection (CECT) and *B. subtilis* 168WT, a kind gift of Dr. Wolfgang Gärtner (MPI for Chemical Energy Conversion, Mülheim a.d. Ruhr, Germany).

First, vegetative bacterial cells were grown in sterile Triptic Soy Broth (*E. coli* and *S. aureus*) or in Brain Heart Infusion broth (*B. subtilis*) at 37°C until an optical density at 600 nm corresponding to 0.4 (*E. coli* and *S. aureus*) or 0.5 (*B. subtilis*). After the incubation period, the cells suspensions were washed three times in PBS by means of centrifugation and resuspension

and then incubated in the dark with the photosensitizer for 30 min at room temperature (*E. coli* and *S. aureus*) or for 90 min at 37°C (*B. subtilis*). Then, photoinactivation experiments were performed as described in references. [6, 7] Briefly, suspensions were placed in 96-wells plates, irradiated with green light for 15 or 30 min (18 and 37 J/cm^2) and serially diluted until 10^{-6} times the original concentration. Colony forming units (CFUs) were counted after 24 h incubation in the dark at 37°C to calculate the survival fraction. Experiments were carried out in duplicate.

For the STED imaging we used a microscopy chamber, with a replaceable coverslip at the bottom, where we dropped a portion of the incubated bacteria suspensions.

Supporting Results

The most relevant photophysical properties of Hyp-apoMb complex were reported in our earlier work. In the following we show additional properties which confirm the existence of a stable 1:1 complex between Hyp and apoMb with useful functional properties.

Figure S1A shows the modeled interaction between Hyp and apoMb, where the photosensitizer occupies the cavity normally hosting the heme. [7] When docked into this hydrophobic cavity, Hyp is protected from aggregation, has reduced rotational freedom resulting in increased fluorescence anisotropy (Figure S1B), and shows photophysical properties similar to those observed for the monomeric dye (Table S1) and for lipid bound Hyp. [8]

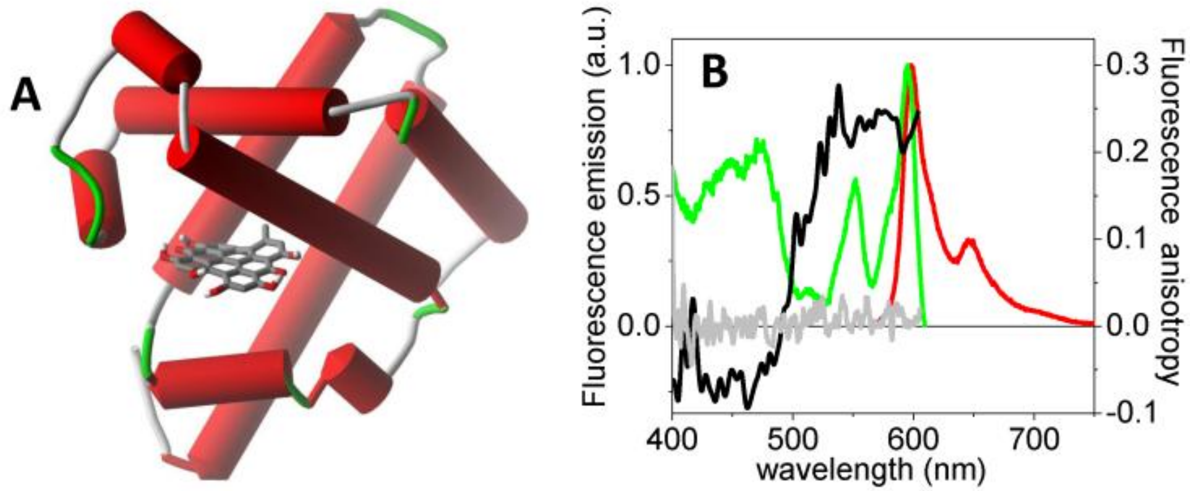

Figure S1. **A.** Docking of Hyp (sticks) into apoMb (cartoon). [7] **B.** Fluorescence emission (red, λ_{ex} = 550 nm), fluorescence excitation (green, λ_{em} = 620 nm) and fluorescence excitation anisotropy (black, λ_{em} = 620 nm) of Hyp-apoMb in PBS solution. Fluorescence excitation anisotropy of Hyp in DMSO (gray, λ_{em} = 620 nm) is reported for reference. T = 25°C.

The triplet state of Hyp when bound to apoMb is shielded from quenching by molecular oxygen, and is thus characterized by a longer lifetime. [7] Figure S2A compares the absorbance change measured at 520 nm [9] after excitation at 532 nm with a nanosecond laser flash for an air equilibrated Hyp solution in DMSO, with that observed for Hyp-apoMb in PBS buffer. The triplet state of Hyp in DMSO decays with a single exponential relaxation with lifetime τ_T = 1.6±0.1 μs. When Hyp is bound to apoMb, the Hyp-apoMb triplet decay occurs with a much longer lifetime, 11.6±0.1 μs in air equilibrated PBS, in agreement with near infrared phosphorescence emission. [7]

Table S1. Photophysical parameters of Hyp and Hyp-apoMb

	Φ_F	τ_F(ns)	τ_T(μs)	Φ_Δ	τ_Δ(μs)
Hyp DMSO	0.35±0.02[10]	5.5±0.1<br>(100%)[11]	1.6±0.1*<br>1.5±0.1[§]	0.28±0.05[§]<br>0.33[5]	5.5±0.1[§]
Hyp-apoMb[7]	0.14±0.02	0.01±0.01<br>(7%)<br>4.0±0.1<br>(25%)<br>6.4±0.1<br>(68%)	11.6±0.1*<br>10.4±0.4[§]<br>10±1#	0.14±0.03[§]	2.4±0.4[§]

Parameters determined in this work: * Laser Flash Photolysis, [§] NIR phosphorescence, [#] FCS.

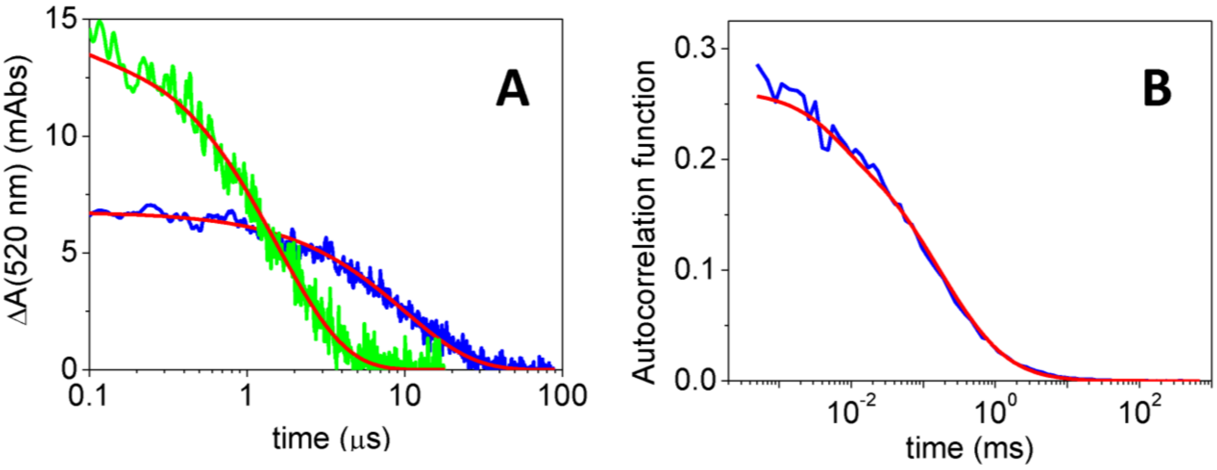

Figure S2. **A.** Triplet-triplet absorbance after photoexcitation with a nanosecond Nd:YAG laser at 532 nm of air equilibrated solutions of Hyp in DMSO (green, 10 μM) and Hyp-apoMb (blue, 10 μM Hyp 30 μM apoMb) in PBS buffer. T = 25 °C. Red lines are the best fits to single exponential decay functions. T = 25 °C. **B.** FCS autocorrelation function of fluorescence emission from a Hyp-apoMb solution (blue, [Hyp] = 10 nM, [apoMb] = 30

μM). The red solid line is the fit to a model for a diffusing species (D = 120±20 $\mu m^2 s^{-1}$) populating also a dark triplet state (τ_T = 10±1 μs). T = 25 °C.

Triplet state kinetics was also confirmed by Fluorescence Correlation Spectroscopy (FCS). Figure S2B shows the autocorrelation function for Hyp-apoMb in a phosphate buffer solution. The signal is best described by a model for a diffusing fluorescent species undergoing formartion of a dark state (triplet state). The retrieved diffusion coefficient is 120±20 $\mu m^2 s^{-1}$, which is perfectly consistent with that of a globular protein with the size of apoMb. The dark state kinetics is characterized by a decay of about 10 μs, in perfect agreement with the triplet-triplet absorption (Figure 2A, blue line), and the near IR phosphorescence emission. [7]

Cell viability

Figure S3 compares the effects of light exposure of *S. aureus*, *B. subtilis*, and *E. coli* after incubation with Hyp-apoMb. While a light dose of 20 J/cm^2 is enough to decrease the bacterial content by 5 log units for Gram-positive *S. aureus*, [7] no sizeable effects are evident on Gram-negative *E. coli*. The case of Gram-positive *B. subtilis* appears intermediate, with only a limited effect of light exposure.

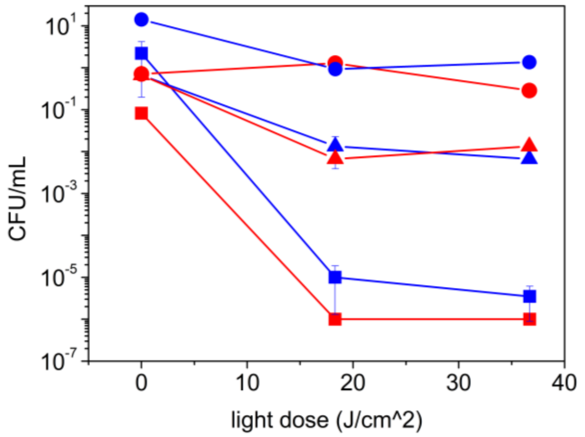

Figure S3. Light dose effects on *S. aureus* (squares), [7] *B. subtilis* (triangles), and *E. coli* (circles) photoinactivation after incubation with Hyp (red) Hyp-apoMb (blue).

References

1. Abbruzzetti S, Bruno S, Faggiano S, Grandi E, Mozzarelli A, Viappiani C. Time-resolved methods in Biophysics. 2. Monitoring haem proteins at work with nanosecond laser flash photolysis. *Photochem Photobiol Sci* **5**, 1109-1120 (2006).

2. Abbruzzetti S, Sottini S, Viappiani C, Corrie JET. Kinetics of proton release after flash photolysis of 1-(2-nitrophenyl)ethyl sulfate (caged sulfate) in aqueous solutions. *J Am Chem Soc* **127**, 9865 - 9874 (2005).

3. Nonell S, Braslavsky SE. Time-resolved singlet oxygen detection. In: *Singlet oxygen, UV-A, and Ozone* (ed^(eds Sies LPaH). Academic Press (2000).

4. Redmond RW, Gamlin JN. A Compilation of Singlet Oxygen Yields from Biologically Relevant Molecules. *Photochem Photobiol* **70**, 391-475 (1999).

5. Losi A. Fluorescence and time-resolved photoacoustics of hypericin inserted in liposomes: dependence on pigment concentration and bilayer phase. *Photochem Photobiol* **65**, (1997).

6. Demidova TN, Hamblin MR. Photodynamic Inactivation of Bacillus Spores, Mediated by Phenothiazinium Dyes. *Applied and Environmental Microbiology* **71**, 6918-6925 (2005).

7. Comas-Barceló J*, et al.* A self-assembled nanostructured material with photosensitising properties. *RSC Advances* **3**, 17874-17879 (2013).

8. Roslaniec M, Weitman H, Freeman D, Mazur Y, Ehrenberg B. Liposome binding constants and singlet oxygen quantum yields of hypericin, tetrahydroxy helianthrone and their derivatives: studies in organic solutions and in liposomes. *J Photochem Photobiol B: Biology* **57**, 149–158 (2000).

9. Michaeli A, Regev A, Mazur Y, Feitelson J, Levanon H. Triplet state reactions of hypericin. Time-resolved laser photolysis and electron paramagnetic resonance spectroscopy. *J Phys Chem* **97**, 9154-9160 (1993).

10. English DS*, et al.* Hypericin, Hypocrellin, and Model Compounds: Primary Photoprocesses of Light-Induced Antiviral Agents. *The Journal of Physical Chemistry A* **101**, 3235-3240 (1997).

11. Yamazaki T, Ohta N, Yamazaki I, Song PS. Excited-state properties of hypericin: electronic spectra and fluorescence decay kinetics. *J Phys Chem* **97**, 7870-7875 (1993).
